# Nonproductive Hepatitis B Virus Covalently Closed Circular DNA Generates HBx-Related Transcripts from the HBx/Enhancer I Region and Acquires Reactivation by Superinfection in Single Cells

**DOI:** 10.1128/jvi.01717-22

**Published:** 2022-12-07

**Authors:** Bo Peng, Zhiyi Jing, Zhongmin Zhou, Yinyan Sun, Guilan Guo, Zexi Tan, Yan Diao, Qiyan Yao, Yi Ping, Xuelei Li, Tengfei Ren, Bin Li, Wenhui Li

**Affiliations:** a Graduate program in School of Life Sciences, Peking University, Beijing, China; b National Institute of Biological Sciences, Beijing, China; c Graduate program in School of Life Sciences, Beijing Normal University, Beijing, China; d Graduate program in School of Life Sciences, Sun Yat-sen University, Guangzhou, China; e Graduate program, Peking Union Medical College, Beijing, China; f Tsinghua Institute of Multidisciplinary Biomedical Research, Tsinghua University, Beijing, China; University of Southern California

**Keywords:** hepatitis B virus, cccDNA, single cell sequence, transcription

## Abstract

Hepatitis B virus (HBV) infection remains a public health problem worldwide. Persistent HBV infection relies on active transcription of the covalently closed circular DNA (cccDNA) in hepatocytes, which is less understood at the single-cell level. In this study, we isolated primary human hepatocytes from liver-humanized FRG mice infected with HBV and examined cccDNA transcripts in single cells based on 5′ end sequencing. Our 5′ transcriptome sequencing (RNA-seq) analysis unambiguously assigns different viral transcripts with overlapping 3′ sequences and quantitatively measures viral transcripts for structural genes (3.5 kb, 2.4 kb, and 2.1 kb) and the nonstructural X gene (0.7 kb and related) in single cells. We found that an infected cell either can generate all viral transcripts, signifying active transcription, or presents only transcripts from the X gene and its associated enhancer I domain and no structural gene transcripts. Results from cell infection assays with recombinant HBV show that nonproductive transcription of cccDNA can be activated by incoming virus through superinfection. Moreover, upon HBV infection, cccDNA apparently can be transcribed in the absence of HBx and produces HBx, needed for productive transcription of other viral genes. These results shed new light on cccDNA transcription at the single-cell level and provide insights useful for improving the treatment strategy against chronic HBV infection.

**IMPORTANCE** Hepatitis B virus (HBV) infection can be effectively suppressed but rarely cured by available drugs. Chronic HBV infection is based on persistence of covalently closed circular DNA (cccDNA) and continuous infection and reinfection with HBV in the liver. Understanding transcriptional regulation of cccDNA will help to achieve permanent transcriptional silencing, i.e., functional cure of HBV. In our study, we found that an infected cell either can generate all viral transcripts, signifying active transcription, or presents only transcripts from the X gene and its associated enhancer I domain and no structural gene transcripts. The nonproductive transcription of cccDNA can be activated by incoming virus through superinfection. Upon an infection, cccDNA apparently can be transcribed in the absence of HBx to produce HBx, necessary for subsequent transcription of other HBV genes. Our studies shed new light on the mechanism of HBV infection and may have implications for a functional cure regimen for HBV.

## INTRODUCTION

Hepatitis B virus (HBV) infects 257 million people worldwide, and infection-associated diseases cause around 1 million deaths annually. Current therapies against HBV suppress but do not cure the infection in the majority of HBV patients (1). An attractive strategy for curative therapy for HBV is to eliminate or permanently silence the molecular reservoir of persistent HBV infection: covalently closed circular DNA (cccDNA). HBV cccDNA is episomal, minichromosome DNA in the cell nucleus formed from the 3.2-kb relaxed circular HBV genome DNA. cccDNA serves as the template for four viral transcripts, known as the 3.5-kb, 2.4-kb, 2.1-kb, and 0.7-kb transcripts (originally characterized by Northern blotting analysis), and there are several copies of cccDNA in HBV-infected cells (2–8). The 3.5-kb transcript either serves as the pregenomic RNA (pgRNA) and is responsible for the HBV core antigen (HBc) and HBV DNA polymerase (Pol) or serves as precore (preC) RNA coding for HBV e antigen (HBe). The 2.4-kb transcript codes for the large envelope protein (L). The 2.1-kb transcript encodes the small (S) and medium (M) envelope proteins. The 0.7-kb transcript codes for the HBV X protein (HBx). Among these, the HBc, Pol, L, M, and S genes are referred to as structural genes (9–13).

The HBV X protein has been shown to control HBV transcription (11, 14, 15); it can induce degradation of the host structural maintenance of chromosomes 5/6 (SMC5/6) complex and functions to promote HBV cccDNA transcription (16). Viral RNAs are actively transcribed from the HBV cccDNA in hepatocytes accompanying a decreased level of SMC5/6 (16, 17). A recent study of HBV-infected, liver-humanized mice revealed transcription-inactive cccDNA induced by treatment of pegylated interferon (peg-IFN) or small interfering RNA (siRNA) in combination with entry inhibition (18). Transcription-inactive cccDNA has also recently been detected in patients who have undergone long-time treatment with nucleos(t)ide analogues (NUCs), as assessed by droplet digital PCR (ddPCR) and/or branched DNA (bDNA) *in situ* analysis (19–21). Still, we only have an incomplete understanding of cccDNA transcription, particularly of quantification of different transcripts and how viral transcription is initiated in individual cells.

Illumination of HBV transcription is constrained by the facts that about two-thirds of the viral genome contains at least two genes and that all HBV transcripts share the same 3′ end. These features have imposed technical challenges for distinguishing different transcripts by conventional analytic approaches. Recently, 5′ rapid amplification of cDNA ends (RACE), which can discriminate hepatitis B viral transcripts, was utilized for profiling HBV viral RNAs in cell cultures and serum from patients (13). Another study used cap analysis of gene expression (CAGE) to generate a map of HBV transcription start sites (TSS) over the entire HBV genome in the liver (22). However, these observations were based on analysis of a mixed population of HBV-infected cells. It is unclear whether there are recognizable patterns of viral transcripts among single cells infected with HBV and whether different cccDNA molecules transcribe autonomously or in a coordinated way. It also remains obscure whether cccDNA can be transcribed in the absence of HBx in single cells at meaningful levels and under what circumstances such an inactive or “latent” cccDNA molecule is activated.

Recent development of single-cell sequencing based on Tn5Prime technology (23) enabled capturing of the 5′ ends of transcripts, including all viral RNAs for sequencing, and subsequent quantification of the whole transcriptome of single cells. In this study, we attempted to exploit 5′ sequencing to quantitatively understand HBV transcription in single hepatocytes. We first established steady HBV infection in liver-humanized FRG (Fah^−/−^ Rag^−/−^/IL-2rg^−/−^ triple knockout) mice and isolated primary human hepatocytes (PHH) from the mice for 5′ single-cell RNA (scRNA) sequencing. We determined the TSS of different genes of HBV and quantified the viral transcripts for pgRNA, precore RNA, and 2.4-kb, 2.1-kb, 0.7-kb, and related RNA in a total of about 2,000 cells. We also used cell infection assays to examine viral DNA and RNA and confirmed with recombinant HBV that nonproductive cccDNA can be activated, in a manner analogous to transactivation, by superinfection of HBV.

## RESULTS

### 5′ sequencing of HBV RNAs reveals the TSS of different genes of HBV in single hepatocytes.

**FIG 1:**
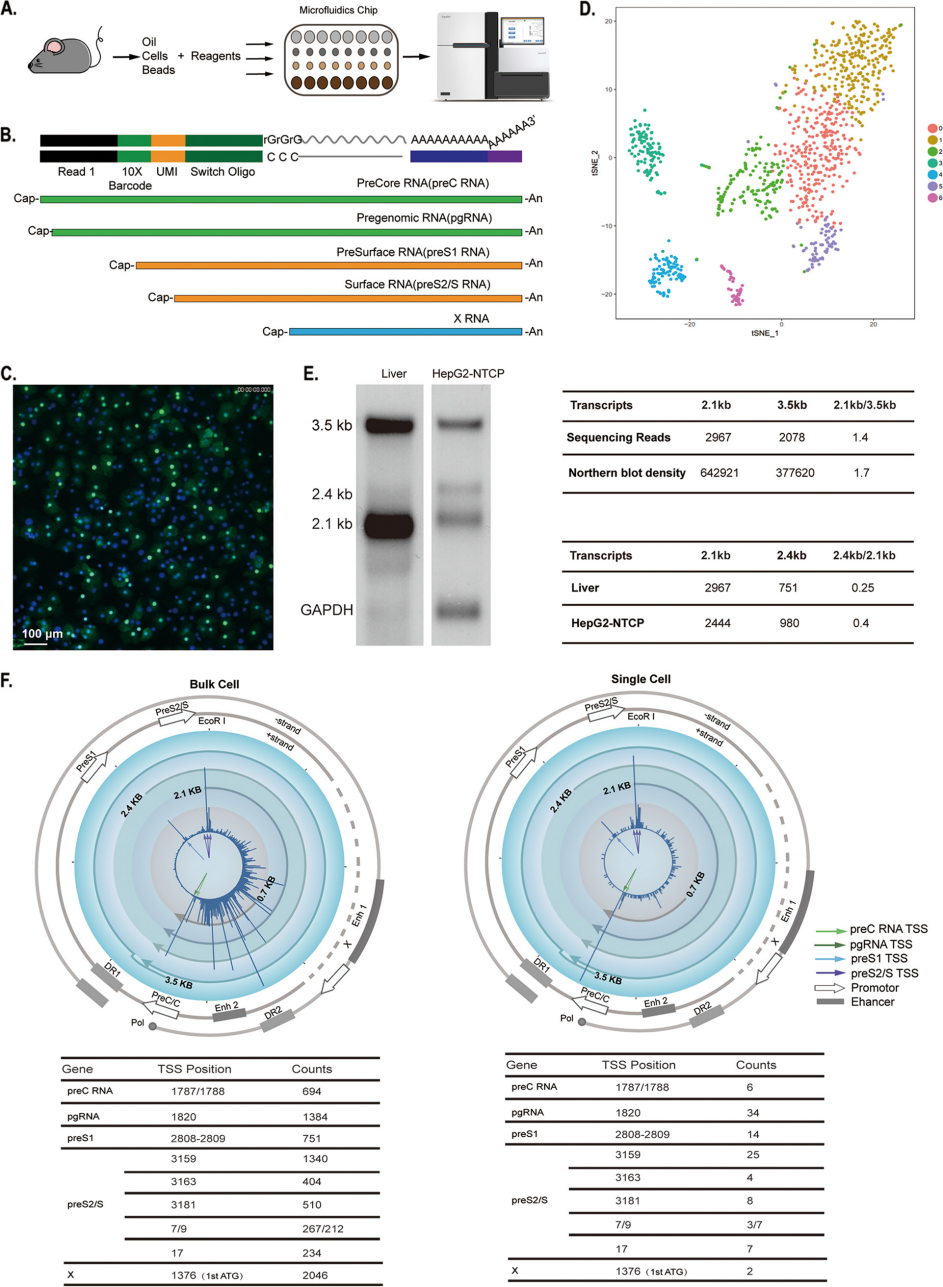
5′ sequencing of HBV RNAs reveals the transcription start sites (TSS) of different genes of HBV in single hepatocytes. (A) Flow chart for 5′ single-cell RNA-seq analysis of HBV-infected liver single hepatocytes. Liver-humanized FRG (hFRG; immunodeficient Fah knockout mice lacking the genes for Rag-2 and the common gamma chain of the interleukin receptor) mice were challenged with HBV (genotype D, ca. 4 × 10^8^ genome copy equivalents); 150 days after infection, human hepatocytes were collected by two-step liver perfusion, isolated as single cells with 10x Genomics chips, and used for generating 5′ single-cell sequence libraries. The libraries were sequenced using HiSeq X Ten, achieving a coverage of ~300,000 reads per cell. (B) (Top) Composition of the HBV transcript library for the 5′ single-cell sequencing. The library has paired-end read 1, which includes the 16-bp barcode used for tagging each individual cell, and a unique molecular identifier (UMI), which serves as a molecular tag to detect and quantify unique mRNA transcripts. (Bottom) Schematic diagram of HBV transcripts (preC RNA, pgRNA, preS1 RNA, preS2/S RNA, and X mRNA) with the same 3′ terminus but a different 5′ end. (C) HBV core antigen in HBV-infected hFRG mice. PHH were isolated from an HBV-infected, liver-humanized FRG mouse. HBV core antigen was stained with 1C10 antibody, in green; nuclei are in blue. (D) t-SNE map of single-cell transcriptomes of PHH isolated from hFRG mouse liver. Human hepatocytes are grouped in populations 0, 1, 2, and 5; other populations (3, 4, and 6) are mouse cells (0: tomato, 1: orange, 2: green, 3: darklyan, 4: deepskyblue, 5: stateblue, 6: violet). (E) (Left) Northern blot analysis of HBV transcripts from humanized liver and HepG2-NTCP cells. (Right) (Top) Ratio of 2.1-kb and 3.5-kb transcripts. For sequencing reads, the 2.1-kb and 3.5-kb RNAs were quantified based on the 5′ sequence. For Northern blot density, the density of the 2.1-kb and 3.5-kb bands detected by Northern blotting was quantified by using ImageJ. (Bottom) Ratio of 2.4-kb and 2.1-kb transcripts. The 2.1-kb and 2.4-kb RNAs were quantified based on the 5′ sequence of HBV-infected humanized liver and HepG2-NTCP cells. GAPDH, glyceraldehyde-3-phosphate dehydrogenase. (F) Circular map for visualization of HBV transcripts in bulk sequencing and one representative single cell. HBV transcripts were identified and quantified by UMI detected by 5′ single-cell sequencing and are labeled in the innermost circle: 3.5 kb for preC (light green arrow) and pregenomic RNA (pgRNA; dark green arrow), 2.4 kb for preS1 RNA (light blue arrow), and 2.1 kb for preS2/S RNA (dark blue arrows). The positions of peaks in the innermost circle correspond to transcription start sites (TSS), the height of the peaks at TSS is proportional to the numbers of corresponding transcripts. The four arrow lines in clockwise orientation show HBV 0.7-kb, 2.1-kb, 2.4-kb, and 3.5-kb transcripts. Open arrows and dark rectangles in the outermost circle show the HBV promoters and enhancers, as indicated. Tables show HBV transcripts in bulk and in a cell. (Left) The count numbers of UMI peaks in total cells for 2.1-kb RNA, 2.4-kb RNA, 3.5-kb preC RNA, and 3.5-kb pgRNA are 2,967, 751, 694, and 1,384, respectively. (Right) There are 331 HBV UMI in the cell among a total of 48,835 UMI, and the count numbers of UMI peaks for 2.1-kb RNA, 2.4-kb RNA, 3.5-kb preC RNA, and 3.5-kb pgRNA are 54, 14, 6, and 34, respectively.

To investigate the HBV transcriptome in single cells, we isolated PHH from liver-humanized HBV-infected FRG mice. We employed a 5′ capture single-cell RNA sequence technology based on Tn*5* transposase; this approach uses the Tn*5* transposase based on the Smartseq2 protocol to create transcriptome sequencing (RNA-seq) libraries (23). It can accurately locate RNA 5′-terminal transcription start sites (TSS) to quantify transcripts with unique molecular identifiers (UMI), making it especially suitable for studying the HBV transcripts, which originate from overlapping viral genomes and cannot therefore be quantified for different transcripts by conventional 3′ sequencing (Fig. 1A and B). Before conducing any sequencing, we first confirmed that the PHH isolated from FRG mice were successfully infected by HBV, evident from the viral HBc protein detected with specific antibody staining (Fig. 1C). The two cDNA libraries were sequenced with a saturation rate greater than 90%, and recovered the median 3,985 host genes per cell and 1,500 to 3,000 hepatocytes in each sample (Fig. 1D). Notably, the ratios of the HBV 2.4-kb and 2.1-kb transcripts and of the 3.5-kb and 2.1-kb transcripts from the sequencing data were comparable to the ratios as assessed using conventional Northern blotting (Fig. 1E), supporting that 5′ sequencing is a reliable approach.

We aligned all of the viral UMI with the HBV genome and used the first nucleotide in the sequencing data as the TSS. By cataloguing the UMI, all HBV transcripts were mapped to the HBV genome and quantified in bulk or individual cells (Fig. 1F, upper portion). To precisely localize the TSS of HBV transcripts, we choose the conventional EcoRI restriction site as a reference position (1) for HBV transcription. 5′ sequencing of both bulk and single cells identified TSS of the 2.4-kb RNA transcript of preS1 at 2808/2809, which is in consistence with previous reports (22, 24, 25); for the 2.1-kb RNA of preS2/S transcripts, different TSS at positions 3159, 3163, and 3181 are indicated (22, 24). The TSS for pgRNA is at 1820, and that for preC RNA is at 1787 and 1788. Among all viral transcripts in bulk cell samples, the preS2/S transcripts are of the highest abundance (51.2%) in all structural genes of HBV, followed by pgRNA (23.8%), preS1(L) (13%), and preC RNA (12%). Notably, the number of transcripts related to the nonstructural X gene (TSS nucleotide [nt] 1376) reaches a level close to that of preS2/S transcripts in bulk cell samples, though it may vary in single cells (Fig. 1F, lower portion).

### Transcripts produced within the HBV enhancer I/X domain in single cells.

**FIG 2:**
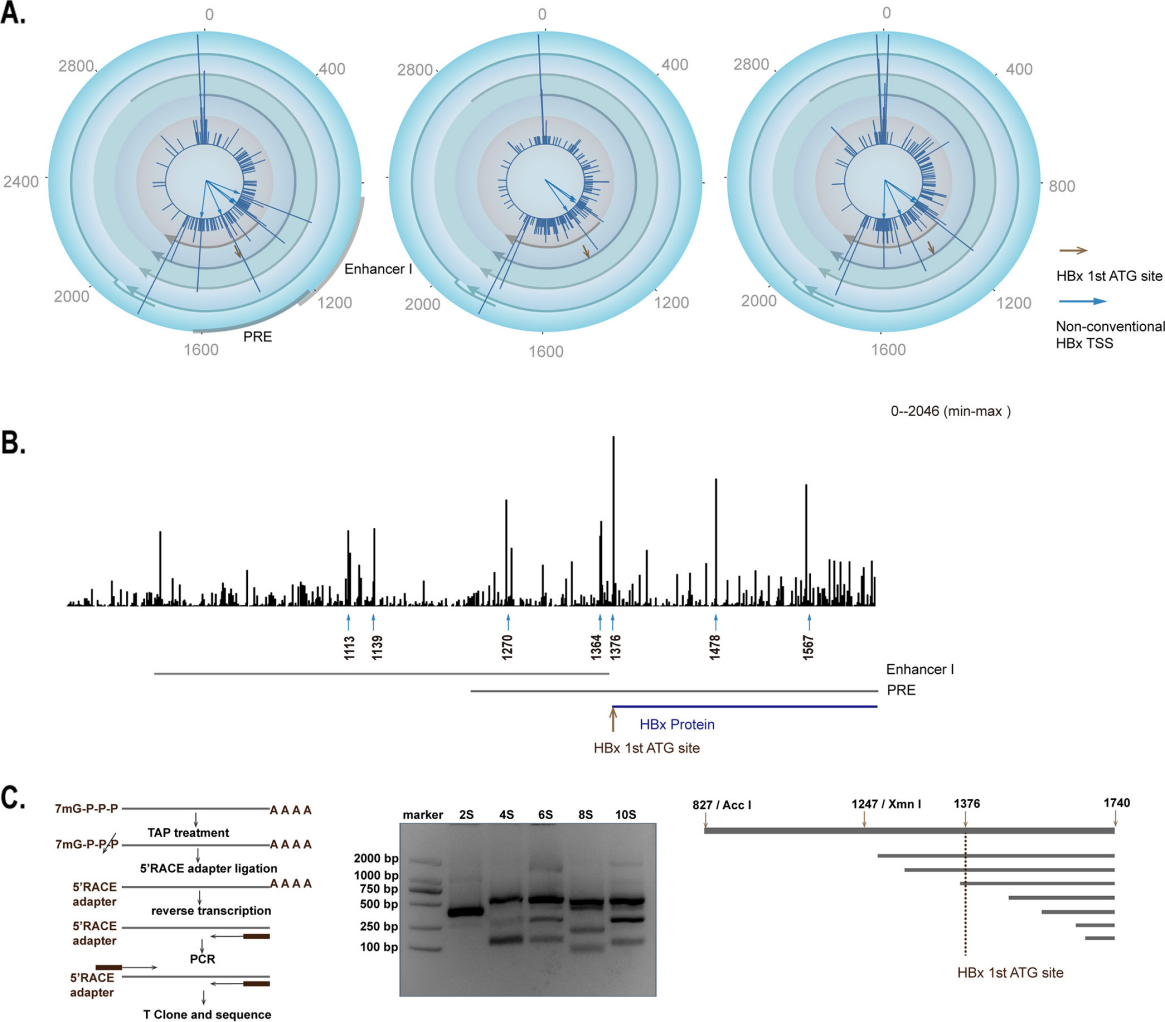
Transcripts produced within the HBV enhancer I/X domain in single cells. (A) Classic and nonconventional HBx TSS in single cells. Three representative single cells showing the classic TSS of HBx transcripts (the position of first ATG of HBx at 1375) (brown arrows) and the most abundant nonconventional HBx TSS peaks (927A, 1113A, 1139G, 1270A, 1376A, 1478G, and 1567A) (blue arrows). HBx TSS are within the region of the enhancer I/X domain. (B) Distribution of the most abundant nonconventional HBx TSS peaks (927A, 1113A, 1139G, 1270A, 1376A, 1478G, and 1567A) (the amount of HBV UMI was >896) from bulk cell cDNA library (blue arrows) and position of the first ATG of HBx protein (brown arrow). The scale shown at the upper-right represents the maximal tag counts. (C) Distribution of HBx TSS based on 5′ RACE analysis. (Left) Flow diagram of HBV full-length 5′ RACE protocol. Total RNA was treated with tobacco acid pyrophosphatase (TAP) to remove the cap structure from full-length mRNA, leaving a 5′-monophosphate. The RNA adapter oligonucleotide was ligated to the RNA population using T4 ligase. The RNAs were reverse transcribed with random decamers. Then, the different-length viral RNAs are amplified by nested PCR. Finally, the PCR fragments were cloned with T-vector and sequenced. (Middle) HBx DNA fragments after nested PCR. HBx cDNAs of different lengths were amplified with different extension times (2 s, 4 s, 6 s, 8 s, and 10 s) by nested PCR. (Right) Alignment of sequenced HBx DNA fragment with HBV genome (from 827 to 1740). The 5′ end of the HBV-specific nested PCR primer is at position 1740.

X gene TSS include the classic one with the first ATG codon at position 1376 and nonconventional ones. Nonconventional TSS of X gene are positioned within the regulatory region including the enhancer I element or posttranscriptional regulatory element (PRE) domain (Fig. 2A and B). In fact, these nonconventional HBx TSS present the most notable trend in the HBV TSS map based on the 5′ sequencing. Briefly, the top TSS peaks detected (the amount of HBV UMI is >896) in a representative sample of bulk cells were 927A, 1113A, 1139G, 1270A, 1364T, 1376A, 1478G, and 1567A (Fig. 2B), and these peaks were able to appear in different combinations in single cells (Fig. 2A). The broad distribution of TSS of HBV X may reflect various lengths of HBx transcripts, which were independently verified by 5′ rapid amplification of cDNA ends (RACE) (Fig. 2C), and they were also identified by two recent studies using 5′ RACE or CAGE (13, 22). However, the exact function, if any, of these transcripts remains unclear, although it is well established that the full-length HBx could substantially enhance HBV transcription (14, 26, 27).

### Coordinated transcription of HBV genes in single cells.

**FIG 3:**
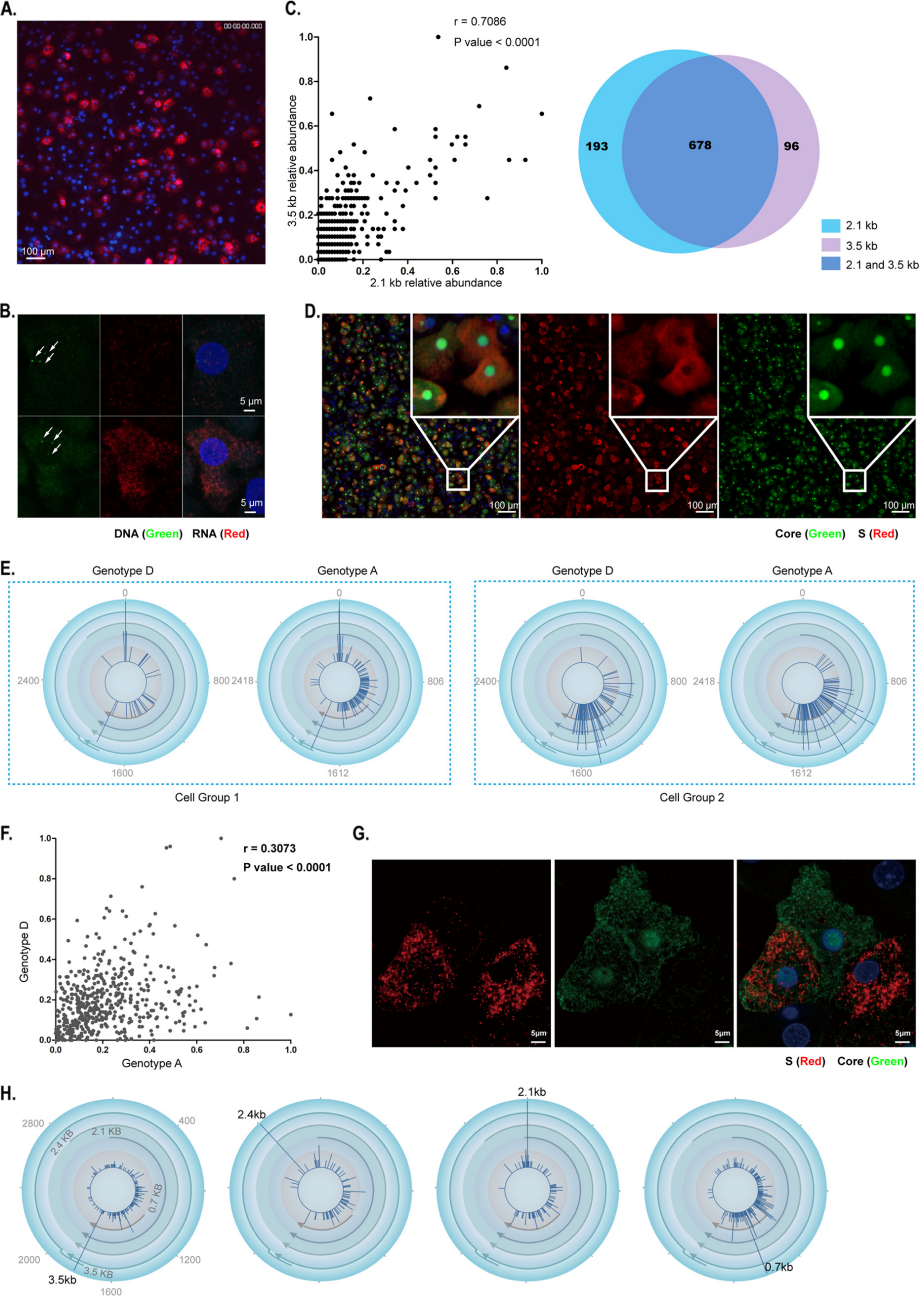
Coordinated transcription of HBV genes in single cells. (A) HBV RNA in HBV-infected hFRG mice. PHH were isolated from an HBV-infected, liver-humanized FRG mouse; total HBV RNA, including structure and nonstructure genes, was labeled in red with a probe covering the whole genome of HBV. (B) FISH analysis of HBV RNA and DNA in single cells. PHH were infected with HBV for 3 days and then were cultured for 4 days in the presence of HAP12 (5 μg/mL) and entecavir [ETV] (10 μg/mL), which inhibit capsid and reverse transcriptase, respectively, hence minimizing the impact of signals from HBV DNA replication intermediates other than cccDNA. HBV-specific bDNA probes targeting the negative strand and the positive strand were used to label HBV DNA and RNA, respectively. Two typical human hepatocytes containing three copies of nuclear HBV DNA are presented, and cccDNA is indicated by arrows. HBV DNA, green; HBV RNA, red; nucleus, blue. (C) Relative amounts of the HBV 3.5-kb and 2.1-kb transcripts in single cells. (Left) UMI number for 3.5-kb and 2.1-kb transcripts in single HBV-infected cells, visualized as dot plots after minimum-maximum normalization (value-minimum/maximum-minimum; UMI number_2.1 kb/cell_ > 1; UMI number_3.5 kb/cell_ > 1). Each dot represents one single cell. (Right) Venn diagram from a quantitative analysis of the numbers of cells that accumulated 3.5-kb transcripts, 2.1-kb transcripts, or both 3.5-kb and 2.1-kb transcripts. (D) Expression of intracellular core and S antigen in single cells. PHH were seeded after isolation from liver of hFRG mice infected with HBV. HBV core antigen was stained with mouse MAb 1C10 (in green), HBV S antigen was stained with mouse MAb 56A1 (in red), and nuclei were stained with 4′,6-diamidino-2-phenylindole (DAPI; in blue). (E) Coordinated transcription of different cccDNA in cells coinfected by HBV genotypes A and D. HBV transcripts (pgRNA, precore, preS1, preS2/S, X, and related RNAs) were quantified and labeled on an HBV circular genome map. Two representative cell groups are shown. (Left) cells with high level transcripts of structure genes; (right) cells with dominant transcripts of X and related RNAs. (F) Dot plots of genotype A and D RNA UMI numbers in single HBV-coinfected cells after normalization (the detailed normalization information can be found in Materials and Methods). The correlation coefficient analysis was conducted using GraphPad Prism (Pearson *r* factor = 0.3073 and *P* value [two-tailed] < 0.0001). Each dot represents one single cell. (G) Representative images of cell expressing both core and S antigen (left bottom), only S (right bottom), or core antigen (upper). HBV core antigen is in green, S is in red, and nuclei are in blue. (H) Disproportional transcripts of HBV in a small subset. HBV transcripts, dominated by one of the four transcripts, were quantified and labeled on circular HBV genome maps. Representative cells with high levels of transcripts of 3.5 kb, 2.4 kb, 2.1 kb, or 0.7 kb are shown individually.

Previous studies of other viruses uncovered transcription heterogeneity of viral genes among individual cells (28–30). We first analyzed HBV viral RNAs by RNA fluorescence *in situ* hybridization (FISH) using probes nondiscriminatory to all viral transcripts, and the FISH imaging study showed that the levels of HBV viral RNA indeed varied among different cells (Fig. 3A). To examine whether the cell-to-cell variation of HBV RNA levels is related to cccDNA copy number, we performed FISH analysis of both HBV DNA and RNA in HBV-infected PHH. We did not observe clear association of RNA levels with cccDNA copies using the FISH method. Actually, HBV RNA levels may vary drastically among cells with the same cccDNA copy number (Fig. 3B). We next took advantage of 5′ scRNA-seq and examined if the relative abundance of specific viral transcripts (measured as UMI) exhibits any notable patterns at the single-cell level. We quantified the 3.5-kb and 2.1-kb transcripts, the two most abundant transcripts among structural genes, and we found that these two genes exhibit considerable consistent expression (Fig. 3C). We also examined the protein products of the 3.5-kb and 2.1-kb transcripts, i.e., the HBV core and S proteins in single cells, by immunofluorescent staining analysis. Consistent with the 5′-scRNA results, about 80% of cells expressed both HBV core and S proteins (Fig. 3D).

To assess whether different cccDNA molecules transcribe autonomously or in a coordinated way within a cell, we infected liver-humanized FRG mice with two different HBV genotypes (genotypes A and D) and then isolated human hepatocytes from the mice for analysis of viral transcripts. Profiling of the HBV transcripts in the single-cell sequence libraries indicated that regardless of the genotype of the infection, different cccDNA molecules within one single cell exhibited highly coordinated transcription (Fig. 3E and F).

Interestingly, although coordinated transcription of HBV genes in single cells presented as the main pattern, small populations of cells presented transcripts disproportionally, e.g., expressing only one of the two viral proteins examined (Fig. 3G) or apparently dominated by one of the four transcripts (3.5, 2.4, 2.1, or 0.7 kb) (Fig. 3H).

### A subset of hepatocytes contained cccDNA-derived HBx-related genes but not viral structural genes.

**FIG 4:**
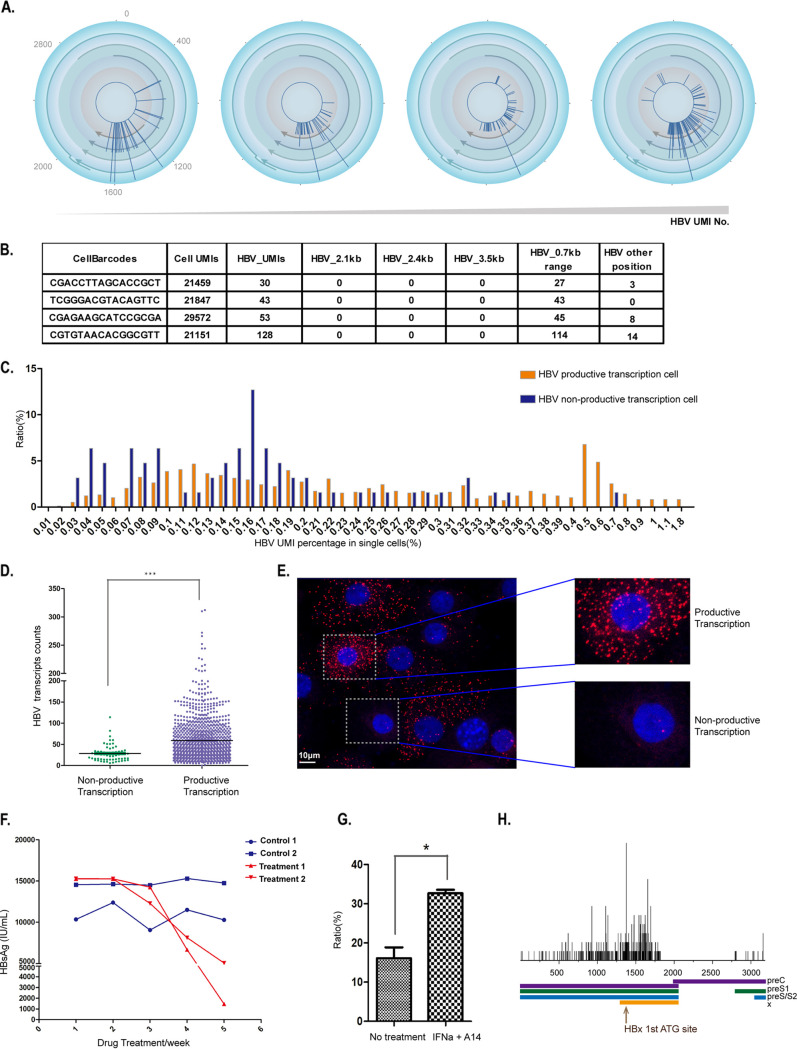
A subset of hepatocytes contains cccDNA-derived HBx-related genes but not viral structural genes. (A and D) Quantification of HBV transcripts from nonproductive transcription, e.g., generation of HBx-related but not viral structure gene-related transcripts in hepatocytes of hFRG mice with steady HBV infection. Cells containing HBV transcripts within the enhancer I/X domain only were analyzed; four representative cells with increasing HBV UMI (from left to right) are shown. The circular maps are depicted as in Fig. 1C. (A) Statistics of HBV X-related transcripts in 1,050 human hepatocytes. Cells were divided into two groups based on the production of HBV transcripts. Sixty-three cells expressed nonstructure HBV gene only (nonproductive transcription). UMI numbers of the nonconventional HBx were quantified at the single-cell level and are shown as individual dots, and the median (shown as horizonal lines) in a nonproductive or a productive cell is 28 or 58, respectively. Each dot represents one single cell. ***, unpaired *t* test *P* value < 0.01 (D). (B) Details of UMI quantification of HBV transcripts (3.5-kb, 2.4-kb, 2.1-kb, and 0.7-kb related) in single cells shown in panel A. Barcodes for the single cells are shown in the first column in the tables, and the cells with corresponding barcodes (from top to bottom) are shown (from left to right) in panel B. (C) Distribution of the productive or nonproductive HBV infection cells with increasing HBV transcript abundance in single cells. The population of HBV-infected cells was divided into 40 parts by HBV UMI percentage in single cells. In each part, the ratio of nonproductive HBV transcription cells (blue columns) and productive transcription cells (red columns) was quantified (in total, 1,050 cells were included; productive cells, *n* = 987; nonproductive cells, *n* = 63) (HBV UMI number ≥ 10). (E) HBV-infected hepatocytes isolated from liver-humanized FRG mice; a human hepatocyte with no productive viral replication is highlighted. HBV-specific probes targeting the negative strand were used to label HBV DNA. Large amounts of HBV progeny were present in the cytosol of productive cells; a typical human hepatocyte is highlighted. HBV DNA, red; nucleus, blue. (F, G, and H). The percentage of HBV nonproductive transcription cells was increased after peg-IFN-α treatment. Peg-IFN-α (50 μg/kg) and HBV entry block antibody A14 (10 μg/kg) were administered to HBV-infected FGR mice two times per week for 4 weeks; PBS was administered as a control. (F) Peg-IFN-α (50 μg/kg) and HBV entry block antibody A14 (10 μg/kg) were administered to HBV-infected FGR mice two times per week for 4 weeks. The serum HBsAg at each week was quantified. (G) Percentage of cells with nuclear but no cytoplasmic viral DNA among all HBV DNA-positive cells. For the no-treatment sample, in total, 111 and 120 HBV DNA-positive cells were imaged, among which 21 and 16 cells contain no cytoplasmic viral DNA from HBV (no HBV replication), respectively. For peg-IFN-α and A14 treatment samples, in total, 134 and 107 HBV DNA-positive cells were imaged, among which 45 and 34 cells contains no cytoplasmic viral DNA from HBV, respectively. *, unpaired *t* test *P* value < 0.05. (H) Details of UMI quantification of HBV transcripts with IFN-α treatment (3.5-kb, 2.4-kb, 2.1-kb, and 0.7-kb related) on linear HBV genome in bulk cells. The position of the first ATG of HBx protein is indicated with a brown arrow.

Recall our data above indicating that a small portion of HBV-infected hepatocytes contained no transcripts for HBV structural genes (i.e., nonproductive) but did express HBx-related RNAs transcribed from the HBV X/enhance I domain (Fig. 4A and B). The UMI numbers for the enhancer I/X domain (HBV X gene-related transcripts) in these cells were at a lower level than the other HBV-infected cells (i.e., the median UMI number was 28 versus 58) (Fig. 4C and D).

To further confirm that there were nonproductive infected hepatocytes, we conducted a DNA FISH assay. We indeed found cells with HBV DNA in the nucleus but not in the cytoplasm (Fig. 4E). Moreover, we carried out an animal experiment to assess such nonproductive infected cells *in vivo*. We treated HBV-infected, liver-humanized FRG mice with an entry blocker (human monoclonal antibody [MAb] 2H5-A14) and pegylated interferon alpha (peg-IFN-α) for 4 weeks, and we monitored the viral marker HBsAg during and after 4 weeks of treatment. The treatment effectively decreased the levels of HBsAg in the treated but not the untreated control animals (Fig. 4F). Consistently, the ratio of nonproductive cells was significantly increased, i.e., to 32% of all infected cells, compared to 16% in the control animals (Fig. 4G). And 5′ RNA-seq analysis of isolated hepatocytes from the mice treated with 2H5-A14/peg-IFN-α showed markedly decreased levels of transcripts for structural genes. Transcripts of X-related RNAs also decreased but presented as the dominant ones among all viral transcripts (Fig. 4H). These results indicate that treatment of entry inhibitor and interferon can effectively inhibit cccDNA transcription, including for X gene and its associated transcripts. Recently, Allweiss et al. reported that similar combinational treatment in liver-humanized mice promotes cccDNA silencing mediated by SMC5/6 (18).

### Host genome-integrated HBV DNA produces a small portion of HBV-related transcripts.

**FIG 5:**
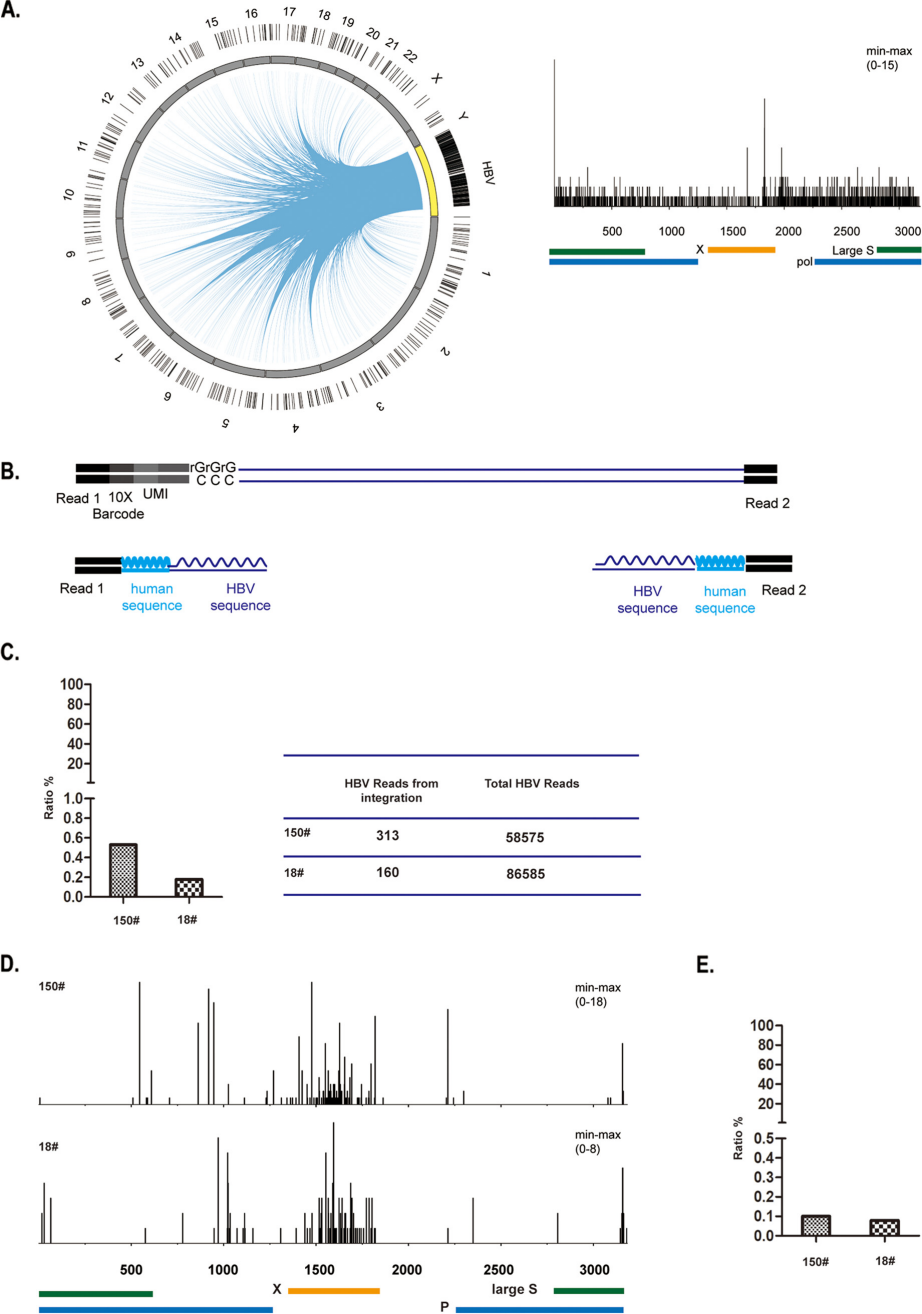
Host genome-integrated HBV DNA produces a small portion of HBV-related transcripts. (A) (Left) Circular map for visualization of integration links between HBV (yellow) and human genome (gray). The outer circle illustrates the density of integration sites on HBV DNA and host genome as indicated. (Right) Integrated breakpoints which include both ends of HBV integration reads at different sites on the HBV genome. A total of 637 HBV integration reads were detected with SurVirus based on the whole-genome sequence (WGS) library. (B) Schematic of representative HBV chimeric reads detected in a 5′ single-cell cDNA library. (C) The ratio of HBV-host chimeric reads in total HBV reads (HBV sequence length in single reads ≥ 20 bp) (left). Two 5′ single-cell cDNA libraries from different mice were analyzed (left, mouse number 150; right, mouse number 18). The amounts of HBV-host chimeric reads and total HBV reads are also shown (right). Totals of 313 and 160 HBV-host chimeric reads were detected among 58,575 and 86,585 HBV reads in two 5′ single-cell cDNA libraries from different mice. (D) Distribution of HBV integrated reads in the HBV genome. The first nucleus acids of all chimeric reads from 5′ single-cell cDNA libraries were quantified and shown in the HBV genome. The scale shown at the upper-right represents the count range (upper, mouse number 150; lower, mouse number 18). (E) The ratio of HBV-host chimeric reads expressed by the enhancer I/X domain in total HBV reads from nonproductive HBV transcription cells. Two 5′ single-cell cDNA libraries from different mice were analyzed (left, mouse number 150; right, mouse number 18).

Previous reports have shown that HBV DNA can integrate into the host genome upon infection (31–34), so beyond cccDNA, another possible source for X-related transcripts is transcription of host genome-integrated HBV DNA. We conducted whole-genome sequencing (WGS) of hepatocytes isolated from liver-humanized FRG mice with HBV infection for 150 days. Consistent with previous reports (31, 32), we found that there were indeed integrated HBV fragments present in the nuclear DNA genomes of these host cells. The HBV integration sites dispersed all chromosomes (Fig. 5A). Quantification of the virus-human fusion transcripts in the single-cell sequence data set indicated that these fusion transcripts comprise only ca. 0.5% of total HBV transcripts (Fig. 5B and C). We did note that the HBV fusion transcripts were mostly from the HBV S or X domain of HBV DNA (Fig. 5D). However, very limited numbers of HBV fusion transcripts expressed by enhancer I/X domain were found in nonproductive HBV transcription cells (Fig. 5E).

Together, these results confirm that the expression of enhancer I/X domain transcripts we consistently detected in the nonproductive HBV-infected hepatocytes occurred from cccDNA. Thus, there is apparently some mechanism that can regulate transcription from specific regions of cccDNA present in the nuclei of nonproductive HBV-infected hepatocytes.

### cccDNA can be transcribed in the absence of the HBx protein.

**FIG 6:**
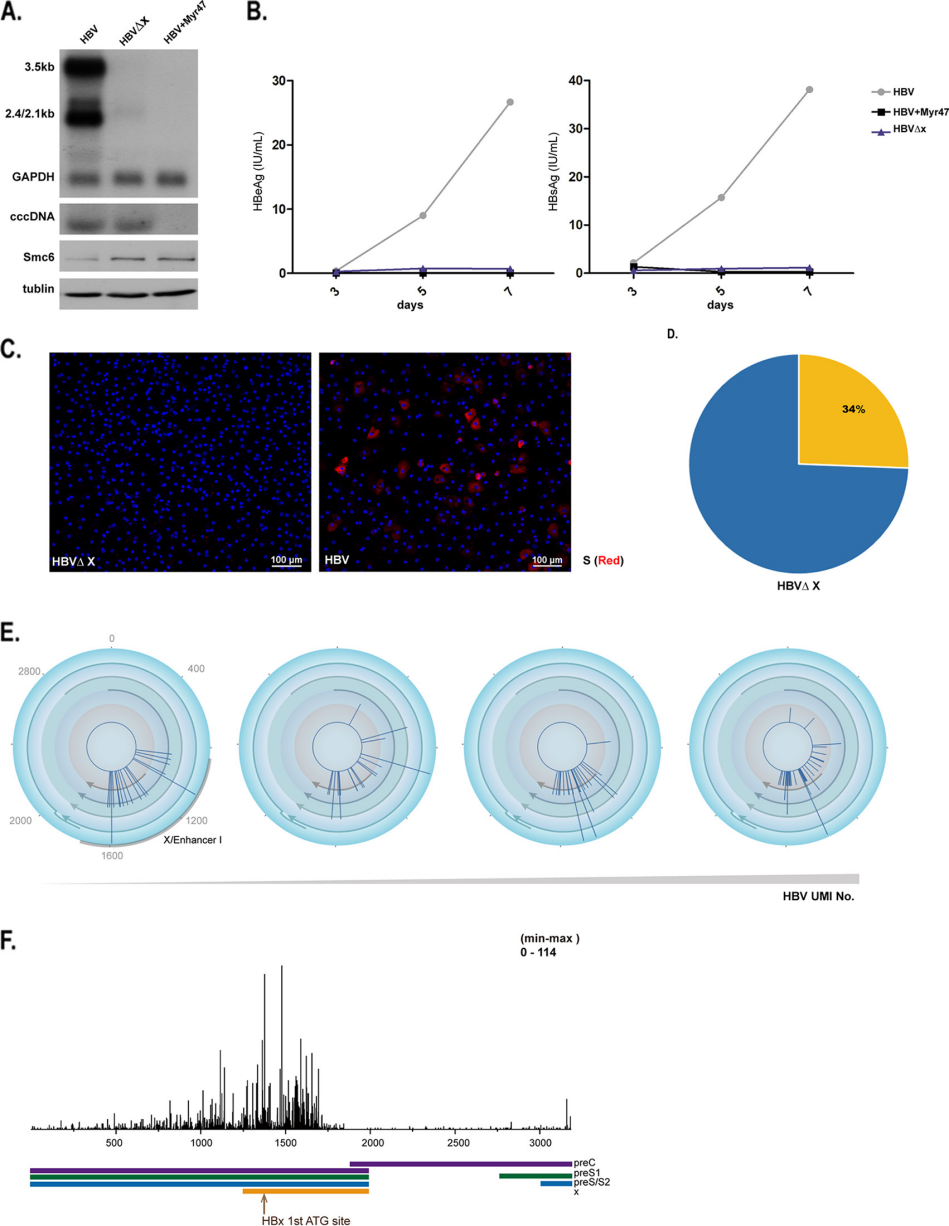
cccDNA can be transcribed in the absence of the HBx protein. (A, B, and C) HBV X protein is essential for HBV productive transcription. PHH were infected for 7 days with HBV-WT or HBV-ΔX, and samples were collected and subjected to Northern blot, Southern blot, and Western blot analyses. PreS1 peptide (Myr47; 200 nM) was included as a control for blocking infection of HBV. The digoxigenin (DIG)-labeled 1.0-copy HBV genome was used as a probe for Northern blot and Southern blot analyses. SMC6 protein was examined by SMC6 antibody (Fitzgerald; 70R-20401). From top to bottom: Northern blot analysis of HBV RNA with GAPDH as a control; Southern blot of cccDNA; Western blot analysis of SMC6 protein with tubulin as a control (bottom) (A). (B) Levels of secreted HBeAg and HBsAg in the supernatants of cells at 3, 5, and 7 days were detected by ELISA, as indicated. (C) HBV S antigen was stained with 56A1 antibody on day postinfection (dpi) 7 (in red); nuclei are in blue. (D) Percentage of HBV-ΔX infected cells. As identified by HBV transcripts with 5′ single-cell sequencing, 766 cells contained HBV UMI among 2,230 cells. (E) Assessment of HBV-ΔX transcription in single cells. PHH were infected with HBV-ΔX *in vitro*. Cells were collected at 7 dpi for 5′ single-cell gene expression analysis. HBV transcripts (pgRNA, precore, preS1, and preS2/S RNA) were quantified and visualized on HBV circular maps. Four HBV-ΔX-infected cells with increasing HBV UMI numbers (from left to right) are shown. (F) Total cell HBV-ΔX transcripts were quantified and visualized on a linear HBV genome. The position of the first ATG of HBx protein is shown (brown arrow). The scale shown at the upper-right represents the maximal tag counts.

The HBV X protein has been shown to control HBV transcription (11, 14, 15). To explore whether RNA can be transcribed from cccDNA in the absence of HBx, we infected PHH with HBV-ΔX virus; for context, the HBV-ΔX genome bears one nucleotide mutation from wild-type HBV (T/C at position 1397), resulting in a stop codon mutation at residue 8 in the HBV X gene. Previous Southern blotting studies showed that cells infected with HBV-ΔX can establish cccDNA (11, 14, 16, 17); however, Northern blotting indicated that the cccDNA in these cells is apparently inactive (i.e., no signal for the targeted viral RNA sequence) (Fig. 6A, B, and C). However, when we conducted 5′-terminal single-cell sequencing analysis of the HBV-ΔX-infected PHH, we detected diminished structural gene expression from HBV-ΔX cccDNA (Fig. 6D and E) and found that there were some HBV transcripts in the HBV-ΔX-infected PHH, the majority of which were from the X/enhancer I domain (Fig. 6E and F).

Together, these results show that at the single-cell level, transcription of HBx and related transcripts may occur irrespective of the transcription of viral structural genes.

### Transcription-inactive cccDNA can be reactivated by superinfection.

**FIG 7:**
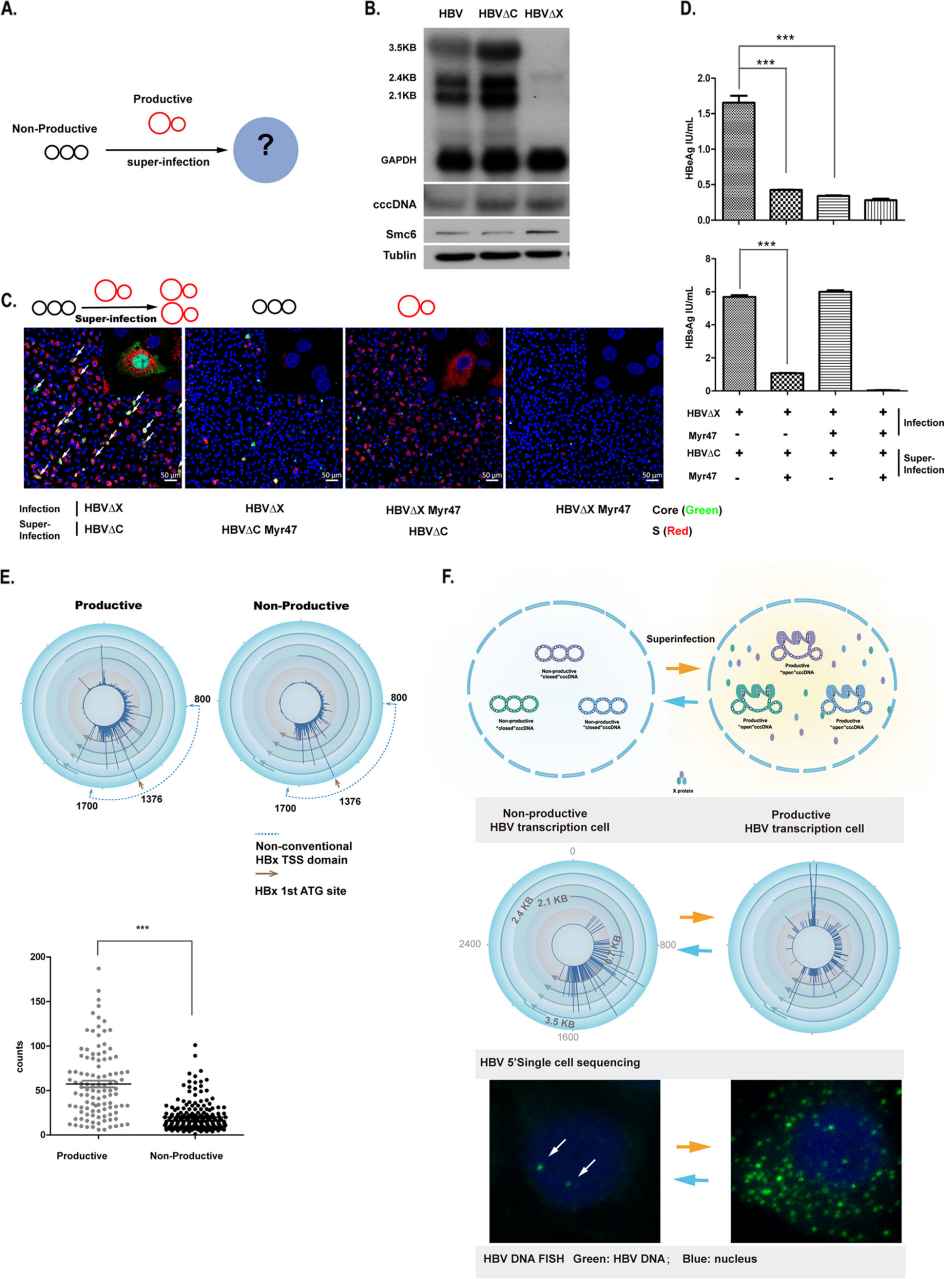
Transcription-inactive cccDNA can be reactivated by superinfection. (A) Strategy of superinfection. HepG2-NTCP cells containing HBV-ΔX cccDNA (nonproductive transcription cccDNA) were superinfected with HBV-ΔC (productive transcription cccDNA). (B) HepG2-NTCP cells were infected for 7 days with HBV-WT, HBV-ΔC, or HBV-ΔX. Samples were collected and subjected to Northern blot, Southern blot, and Western blot analyses as for Fig. 6B. From top to bottom: Northern blot analysis of HBV RNA with GAPDH as a control, Southern blot of cccDNA, and Western blot analysis of SMC6 protein with tubulin as a control (bottom). (C) Superinfection of HBV-ΔC activates HBV-ΔX expression of core protein. HepG2-NTCP cells were infected with HBV-ΔX, followed by superinfection of HBV-ΔC on dpi 3 of the primary HBV-ΔX infection. HBV core antigen was stained with 1C10 antibody on dpi 14 (in green), and HBV S was stained with 56A1 antibody (in red); nuclei are in blue. PreS1 peptide (Myr47; 200 nM) was included as a control for blocking primary infection of HBV-ΔX, blocking secondary superinfection of HBV-ΔC, or blocking both primary infection and superinfection. In all three cases, little or no expression of core protein by HBV-ΔX was observed. (D) Levels of secreted HBeAg and HBsAg in the supernatants of cells were detected by ELISA, as indicated. ***, unpaired *t* test *P* value < 0.05. (E) Active transcription of HBx transcripts is required for HBV productive transcription. (Left) Quantification of transcripts of productive transcription (structure gene UMI > 10) and nonproductive transcription (nonstructure gene UMI > 10). Cells from HBV-infected hFRG mouse liver were assessed with 5′ single-cell gene expression analysis. The transcripts were quantified and visualized on an HBV circular map based on HBV UMI. The first ATG site in HBV X mRNA is highlighted with a brown arrowhead in the maps; the nonconventional X mRNA TSS domain is indicated with a blue dotted line. (Right) Quantification of the classic X transcripts (covering HBV full-length X protein coding DNA sequence [CDS]) in all X-related transcripts (TSS within 800 to 1,700 nucleotides in the HBV genome) in single cells from two cell populations. (F) Working model of cccDNA transcription. In productively infected cells, cccDNA molecules may be in an open conformation and are active and capable of structural gene transcription, while in cells with nonproductive infection, cccDNA molecules may be in a closed conformation and only transcribe in the domain of enhancer I/X. Various-length transcripts related to the X gene are generated even in cells with nonproductive infection.

To assess whether nonproductive HBV transcription from cccDNA can be reactivated, we conducted cell culture experiments in which we infected HepG2-NTCP cells with different recombinant viruses: wild-type HBV (HBV-WT); HBV-ΔX, which is transcriptionally inactive upon infection and does not express core or S protein; and HBV-ΔC, which is transcriptionally active upon infection and expresses S but not core protein (Fig. 7A and C). HepG2-NTCP cells infected with the recombinant viruses produced viral RNAs in the pattern expected: HBV-WT and HBV-ΔC infection generated all viral RNAs, while HBV-ΔX infection generated much-diminished transcripts as assayed by Northern blotting (Fig. 7B). Importantly, when HepG2-NTCP cells already infected with HBV-ΔX were inoculated with HBV-ΔC (i.e., superinfection), 8% of cells became core positive 7 days after the superinfection, while no cells were core positive without HBV-ΔC superinfection (Fig. 7C). Enzyme-linked immunosorbent assay (ELISA) of supernatant also showed that HBeAg levels were markedly increased in cultures successfully infected with HBV-ΔC but not those blocked by the entry inhibitor (Myr47) (Fig. 7D). These results show that expression of the HBx in response to superinfection can effectively rescue HBV infection arrested by deficient X gene expression in the same cells, indicating that cccDNA in such a nonproductive HBV infection is not permanently silenced; rather, it can be readily reactivated. Finally, we quantified X transcripts with TSS at or before 1376A, which correspond to the full length of the HBx protein. Such transcripts are generated at higher frequency in cells with productive infection than in those under nonproductive infection (Fig. 7E), supporting the notion that the protein but not mRNA of X is essential for active transcription of HBV structural genes.

## DISCUSSION

In this study, by using 5′ RNA-seq, which can effectively differentiate and quantify all HBV transcripts (pgRNA, precore, preS1, preS/S2, and X) in single cells, we showed that a group of hepatocytes transcribe HBx-related but not viral structural genes. Our HBV DNA FISH analysis confirmed that no progeny viruses were produced in these nonproductive infected hepatocytes. This group of cells comprised small percentage (~6%) of hepatocytes isolated from liver-humanized mice stably infected with HBV, but they may represent an important reservoir of chronic infection, in which the virus may be at a stage analogous to latency but is capable of subsequent reactivation. We tested this hypothesis by a set of *in vitro* infection assays with recombinant viruses, and we proved that cells containing HBV with nonproductive transcription can be reinfected and the prior latent HBV infection can be effectively reactivated by new incoming virions (Fig. 7F). In line with this, Allweiss et al. recently showed that treatment of peg-IFN resulted in SMC5/6-mediated cccDNA silencing in single cells, but maintenance of the suppression required blockage of reinfection in liver-humanized mice (18).

In the clinic, three recent studies of single hepatocytes from patients showed that a longer duration of NUC treatment is usually associated with a greater proportion of infected hepatocytes with nonactive viral transcription (19, 21, 35). However, discontinuation of NUC treatment leads to viral rebound in most patients, even they have been on treatment for years and have no quantifiable HBV in blood prior to treatment cessation. Currently, it would be difficult to measure viral infection rebound at the single-cell level in these patients, but it is reasonable to speculate that progeny viruses amplified by and disseminated from small numbers of cells with productive viral transcription will easily lead to infection and superinfection of hepatocytes in liver and eventually evident viremia in the blood.

At the cellular level, HBV X protein is known to be a key factor controlling HBV transcription (14, 17, 36). The present study showed that it is also required for HBV reactivation in single cells. In theory, both cccDNA and viral integrants in-frame fused with a host open reading frame (ORF) may generate X protein. Our sequencing data revealed that only very small proportion of X transcripts (less than 0.5%) were derived from integrant-X DNA and the vast majority of X transcripts were from cccDNA, including those that had undergone nonproductive transcription. It has been obscure whether cccDNA could be transcribed in the absence of HBx itself. Our results indicate that transcription of HBx-related RNAs appears to be randomly initiated along a region covering the HBx coding sequence as well as the HBV X/enhancer I domain, and it can be expected that in cells infected with HBV-WT or a mutant, generation of HBx and related transcripts occurs spontaneously, although the possibility of transfer of X transcripts within exosomes in initial infection cannot be completely excluded. Productive transcription of viral structural genes apparently follows accumulation of transcripts related to the X gene. Presumably, after binding to the enhancer I/X domain, host RNA polymerase II synthesizes X transcripts in a stochastic manner at different TSS and this would generate HBV X transcripts of different lengths; HBx transcripts incapable of translating to HBx protein cannot support productive cccDNA transcription. Accumulation of X transcripts, in particular those capable for translation of functional X protein (which can lead to degradation of host SMC5/6), is required to spark cccDNA productive transcription.

In patients under long-term NUC treatment, viral amplification and spread are suppressed at a low level. However, quasispecies of HBV, which are commonly seen in patients (37, 38), can support cross-activation of cccDNA within single cells and hence production of infectious virus even if the original viruses are defective. Therefore, in addition to virus produced by aforementioned productive cells, nonproductive infected cells may also contribute to maintenance of continuous infection in the liver. Disruption of this cycle, e.g., blockage of infection and reinfection of hepatocytes, may present an important gap to be filled for a more effective HBV regimen.

## MATERIALS AND METHODS

### Isolation and culture of PHH.

Liver-humanized FRG mice were housed in a biosafety level 2 (BSL2) animal facility at the National Institute of Biological Science (NIBS), Beijing. All studies were performed in accordance with institutionally approved protocols and adherence to guidelines of the National Institute of Biological Sciences guide for the care and use of laboratory animals. Primary human hepatocytes (PHH) were obtained from liver-humanized FRG mice with two-step perfusion as previously described (39).

### PHH.

PHH were purchased from Shanghai RILD Inc. Cells were seeded in hepatocyte culture medium (HCM) medium with 10% fetal bovine serum (FBS) and maintained in primary hepatocytes medium (PMM) as previously described (39).

### Cell lines.

Human hepatocellular carcinoma cell line Huh-7 was obtained from the Cell Bank of Type Culture Collection, Chinese Academy of Sciences. Human hepatocellular carcinoma cell line HepG2-NTCP(AC12) was established in-house and maintained as previously described (40, 41).

### Viruses.

**(i) HBV.** HBV virions were produced by transfection of Huh-7 cells with plasmids containing 1.05 copies of cDNA of genotype D HBV under the control of a cytomegalovirus (CMV) promoter as previously described. Production of HBV genotype A followed the genotype D protocol.

**(ii) HBV antigen-deficient virus.** HBV mutation virions were produced by transfection of Huh-7 cells with a plasmid containing 1.05 copies of the HBV mutation and its complementary ORF plasmid at a ratio of 3:1 as described above the mutants were named HBV-ΔX and HBV-ΔC. The HBV mutation plasmids were constructed by altering the X ORF 8th and core ORF 38th amino acid codons from ATG to a stop codon. The changes do not interfere with other HBV ORF coding. The complementary plasmids encoding X or core are under the control of a CMV promoter.

### Antibody.

56A1 is a mouse MAb (IgG1) recognizing HBV S protein. 1C10 is a mouse MAb specific for HBV core protein.

### Quantification of HBV genome equivalent copies.

HBV copies were examined as previously described (42). HBV genotype D DNA was quantified by primers F-GGAAGAGAAACCGTTATAGAG and R-CCTGCCTCGTCGTCTAACAAC. HBV genotype A DNA was quantified by primers F-GGAAGAGAGACGGTACTTGAA and R-TCGGTCCCGTCGTCTAACA. The viral genome equivalent copies were calculated based on a standard curve generated with known copy numbers. Real-time quantitative PCR (qPCR) experiments were performed using a SYBR Premix *Ex Taq* kit on a Bio-Rad real-time system instrument.

### PHH single-cell mRNA sequence cDNA library construction.

Perfused PHH from liver-humanized mice were sequenced using the Chromium single-cell 5′ gene expression platform (10x Genomics). Approximately 3,000 PHH from each sample were directly loaded into each sample well following manufacturer instructions and combined into droplets with barcoded beads using the Chromium controller. Manufacturer specifications were followed for generation of the barcoded libraries, and then the samples were sequenced to an average depth of 300,000 reads per cell on an Illumina sequencer (HiSeq X Ten).

### Bioinformatics analysis of 5′ single-cell expression of PHH.

Sequenced samples were processed using the Cell Ranger 2.1 pipeline and aligned to the GRCh37 (hg19) human reference genome, GRCm38 (mm10) mouse reference genome, and reference genome sequences of genotype D (GenBank accession number U95551.1) and genotype A. A digital gene expression matrix (DGE) was generated containing the raw UMI counts for each cell in a given sample. Original data in the study are available in the NCBI database (NCBI tracking system number 21176677; GSE: 156690) (https://www.ncbi.nlm.nih.gov/geo/).

### HBV infection of HepG2-NTCP cells or PHH and HBV antigen detection.

A total of 1 × 10^7^ genome equivalent copies of HBV or HBV antigen-deficient virus supplemented with 4% polyethylene glycol 8000 (PEG 8000) were incubated with 2 × 10^4^ HepG2-NTCP cells or PHH. The cells were maintained for 7 days subsequently in PMM supplemented with 2% FBS and changed to fresh medium every 2 days. For single-cell sequencing, the cells were collected after trypsin digestion. For antigen staining, the intracellular horseradish peroxidase was removed with phosphate-buffered saline (PBS) supplemented with 3% H_2_O_2_ and 40% CH_3_OH after the cells were fixed with 3.7% paraformaldehyde (PFA) and then stained for core with 1C10 Ab and for S with 56A1 Ab, followed by the phycoerythrin (PE) tyramide signal amplification (TSA) fluorescence system. The cell images were captured with a Nikon structured illumination microscope (SIM) or PerkinElmer spinning-disk microscope. Secreted viral antigen HBsAg and HBeAg were detected by ELISA.

### HBV superinfection of HepG2-NTCP cells.

A total of 1 × 10^5^ cells were inoculated with 500 genome equivalent copies/cell of one virus in the presence of 4% PEG 8000. Three days after the primary virus infection, these cells were treated with 10% FBS for 2 days and then were superinfected by inoculation of 500 genome equivalent copies/cell of another different recombinant HBV. Subsequent culture steps were the same as for monoinfection.

### HBV infection or different genotypes of HBV coinfection in an hFRG mouse model.

Humanized FRG (hFRG; Fah^−/−^ Rag^−/−^ IL-2rg^−/−^ triple knockout) mice were transplanted with human hepatocytes. hFRG mice with a human serum albumin level around 2 mg/mL were used for challenge with one or more genotypes of HBV. HBV was produced by Huh-7 cell transfection.

**(i) Monoinfection.** Three hFRG mice were challenged with 4 × 10^8^ genome equivalent copies of HBV in 200 μL per mouse by orbital injection. Body weight and serum HBV DNA copy numbers of each mouse were monitored every week. For single-cell sequencing, cells were isolated by liver perfusion when the blood HBV titer reached about 1 × 10^9^ to 9 × 10^9^ copies/mL. Intracellular core antigen or S antigen was detected by specific antibody in perfused PHH seeded on culture plates.

**(ii) Coinfection.** Two hFRG mice were inoculated with 1 × 10^9^ genome equivalent copies of HBV genotypes A and D in 200 μL per mouse by orbital injection, following a protocol similar to that for monoinfection in FRG mice. DNA levels of secreted different genotypes of HBV were measured periodically from serum samples with specific primers. HBV genotype A- and D-coinfected mice were sacrificed when the serum genotype A DNA level reached about 6 × 10^9^ copies/mL and the genotype D DNA level reached about 4 × 10^8^ copies/mL.

### HBV transcript mapping.

The reads mapped to HBV genotype A and D genomes were selected by their genomes, and the UMI counts from each specific HBV genome were calculated. By using the cell barcodes of these reads, they were further assigned to individual cells. Moreover, HBV UMI transcribed from a specific genome region can also be counted according to the position to which they mapped. The amounts of the 3.5-kb, 2.4-kb, and 2.1-kb transcript species of HBV in each cell were also calculated according to their transcription start sites. For HBV genotype D (GenBank accession number U95551.1), the start sites of the 3.5-kb, 2.4-kb, and 2.1-kb transcripts are within position 1741 to ~1869 bp, 2791 to ~2850 bp, and 3040 to ~61 bp, respectively. For HBV genotype A, the start sites of the 3.5-kb, 2.4-kb, and 2.1-kb transcripts are within position 1739 to ~1867 bp, 2795 to ~2854 bp, and 3077 to ~59 bp, respectively. Six transcription start sites which were most frequently used within the genotype D HBV transcripts of 3.5 kb, 2.4 kb, and 2.1 kb were annotated (position 1787, 1820, 2808, 3159, 3163, and 3181 bp). HBV UMI transcribed from every single position of HBV genome in single cells were also calculated for visualization with Circos. For a group of selected cells or all cells that passed the quality filter, the total HBV UMI amounts from every single position of HBV genome were also calculated.

### t-SNE plots of gene expression.

Seurat R package (version 2.3.4) was used for quality control and dimensionality reduction analysis. Cells with mitochondrial gene proportions over 50% were discarded. During dimensionality reduction analysis, we first identified highly variable genes across the single cells, after controlling for the relationship between average expression and dispersion. Centered and scaled UMI count values per cell were transformed by multiplying by 10,000, adding 1, and then taking the log of these values. These values were plotted using a custom R script and ggplot2 with the YlOrBr scale from the RColorBrewer package.

### Analysis of HBV DNA integrations and HBV-host chimeric transcripts.

The next-generation sequencing (NGS) data sets used in the project were collected from samples of two liver-humanized FRG mice (mouse numbers 18 and 993). The library was made following the instruction of NEXTflex rapid DNA-seq kit (Bioo Scientific). Whole-genome DNA paired-end sequencing data were collected by using the Illumina HiSeq platform. The average coverage was 100×. The HBV-host chimeric DNAs were predicted by SurVirus (43).

For detecting HBV-host chimeric RNA transcripts, data were obtained from two single-cell 5′ cDNA libraries of liver-humanized FRG mouse samples (mouse numbers 18 and 150). Single-cell sequencing reads successfully mapped to the HBV genome, but for soft clippings of more than 30 bp, their mapping results were collected for analysis. These >30-bp-long clippings were then cut and mapped to the human genome to find whether clippings can match human genome with more than 30 bases. All those reads that mapped to both HBV and human genomes with more than 30 bases were collected as preliminary candidates of HBV-host integrants. The host reference is Homo sapiens genome assembly GRCh38, while the virus reference data set is U95551.1.

### Material availability.

Plasmids generated in this paper will be provided upon request and with an material transfer agreement (MTA).

### Data and code availability.

The data sets generated during this study are available at the NCBI database (NCBI tracking system number 21176677; GSE156690). The code supporting the current study has not been deposited in a public repository but is available upon request.
